# Proactive cursorial and ambush predation risk avoidance in four African herbivore species

**DOI:** 10.1002/ece3.11529

**Published:** 2024-06-05

**Authors:** Emily Bennitt, Hattie L. A. Bartlam‐Brooks, Tatjana Y. Hubel, Neil R. Jordan, John W. McNutt, Alan M. Wilson

**Affiliations:** ^1^ Okavango Research Institute, University of Botswana Maun Botswana; ^2^ Structure and Motion Laboratory Royal Veterinary College Hatfield UK; ^3^ Wild Entrust Maun Botswana; ^4^ Centre for Ecosystem Science, School of Biological, Earth and Environmental Sciences University of New South Wales Sydney New South Wales Australia; ^5^ Taronga Institute of Science and Learning, Taronga Conservation Society Syndey New South Wales Australia

**Keywords:** antelope, carnivore, predator–prey, risk avoidance, ungulate

## Abstract

Most herbivores must balance demands to meet nutritional requirements, maintain stable thermoregulation and avoid predation. Species‐specific predator and prey characteristics determine the ability of prey to avoid predation and the ability of predators to maximize hunting success. Using GPS collar data from African wild dogs, lions, impala, tsessebes, wildebeest and zebra in the Okavango Delta, Botswana, we studied proactive predation risk avoidance by herbivores. We considered predator activity level in relation to prey movement, predator and prey habitat selection, and preferential use of areas by prey. We compared herbivore behaviour to lion and wild dog activity patterns and determined the effect of seasonal resource availability and prey body mass on anti‐predator behaviour. Herbivore movement patterns were more strongly correlated with lion than wild dog activity. Habitat selection by predators was not activity level dependent and, while prey and predators differed to some extent in their habitat selection, there were also overlaps, probably caused by predators seeking habitats with high prey abundance. Areas favoured by lions were used by herbivores more when lions were less active, whereas wild dog activity level was not correlated with prey use. Prey body mass was not a strong predictor of the strength of proactive predation avoidance behaviour. Herbivores showed stronger anti‐predator behaviours during the rainy season when resources were abundant. Reducing movement when top predators are most active and avoiding areas with a high likelihood of predator use during the same periods appear to be common strategies to minimize predation risk. Such valuable insights into predator–prey dynamics are only possible when using similar data from multiple sympatric species of predator and prey, an approach that should become more prevalent given the ongoing integration of technological methods into ecological studies.

## Graphical abstract

In most ecosystems, prey must avoid capture by multiple species of predator. Prey minimizes movement and avoids high‐use areas when top predators are most active. Considering multiple species of predator and prey is advantageous in ecology.

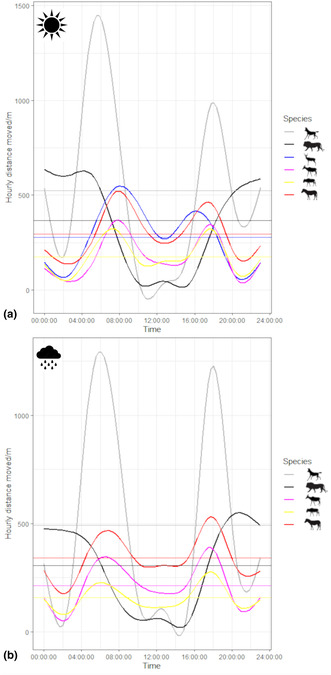

## 1 INTRODUCTION

Prey and predators usually exist in complex multi‐species systems that lead to competition within guilds (Radloff & Du Toit, 2004) and an arms race between them (Wilson et al., 2018), with predators striving to catch prey while prey seek to escape. Herbivores need to meet their nutritional requirements while minimizing predation risk and, in most systems, allowing optimal thermoregulation (Veldhuis et al., 2020). Such trade‐offs between risks and rewards are harder to manage during periods of resource scarcity, such as the dry season in African savanna systems, when prey may endure higher predation risk to meet their resource requirements (Palmer et al., 2017). Predation risk includes the risks of encounter, attack and kill (Creel, 2018), all of which vary spatially and temporally (Kittle et al., 2022), and differ between predator–prey species dyads (Rigoudy et al., 2022). Ambush predators seek high encounter rates with prey in habitats allowing close approaches (Christianson et al., 2018), so, when hunting, can be linked to habitats conducive to hunting success (Donadio & Buskirk, 2016), leading to long‐term predation risk for prey in those areas (Droge et al., 2017). Cursorial predators often cover large areas and are less predictable in their movements (Courbin et al., 2019), so tend to create periods of short‐term predation risk for prey (Droge et al., 2017). Predation risk can lead to non‐consumptive effects on prey behaviour, movement patterns and ultimately population demographics (Middleton et al., 2013). Overall, ambush predators tend to elicit more extreme non‐consumptive effects than cursorial species (Middleton et al., 2013; Say‐Sallaz et al., 2023), and these effects are most apparent for smaller‐bodied herbivores (Moll et al., 2016).

Prey species display adaptive reactions to different predation threats: generally, prey species react with vigilance to ambush predators and evasion when confronted by cursorial species (Palmer & Packer, 2021). Prey species with different physiologies and body masses vary in their ability to adapt activity patterns to predation risk alone (Moll et al., 2016); such adaptations may vary with seasonal changes in forage availability and resource requirements (Palmer et al., 2017). Large‐bodied prey such as plains zebra (*Equus quagga*) can readily move away from predators (Fischhoff et al., 2007), but, as hind–gut fermenters, they need to feed for longer periods than ruminants to reach their nutrient requirements so are less able to alter their temporal foraging patterns (Traill et al., 2016), although, unlike ruminants, zebra can compensate for low forage quality by increasing the amount of forage consumed (Schwarm et al., 2009). Zebra are forage generalists, requiring bulk intake, whereas smaller species such as blue wildebeest (*Connochaetes taurinus*) are more specialized, so might be more disadvantaged by moving away from a risky but productive area and may show a less extreme reaction to predation risk (Martin & Owen‐Smith, 2016).

Predator activity levels, which can be derived from hourly distance moved (Hayward & Slotow, 2009), inform predation risk and vary with time of day and associated conditions, such as visibility and temperature (Suraci et al., 2022), whereby predators tend to engage in low‐activity behaviours such as resting during the hot, middle of the day and undertake high‐activity behaviours such as patrolling and hunting when temperatures are cooler (Hayward & Slotow, 2009). Predators may select different habitats based on their behaviour (Courbin et al., 2016), meaning that predation risk can vary with habitat and predator activity, with the latter being linked to time of day (Hayward & Slotow, 2009). Prey selection differs with environment and time of day based on prey abundance and catchability (Gehr et al., 2018), so predation risk can be highly dynamic, both spatially and temporally. Timing and predator activity levels should therefore be considered when predator–prey dynamics are analysed (Suraci et al., 2022), multiple species of prey and predator should be included in studies whenever possible (Say‐Sallaz et al., 2019) and use of biologging technology is advantageous (Suraci et al., 2022). Very few studies record similar data from multiple species of predators and prey in the same environment (Suraci et al., 2022), so the incorporation of these factors into a comprehensive study is rare and can uncover information on proactive predation avoidance behaviour that would otherwise not be detected.

In southern Africa, numerous species of predator and prey with differing characteristics occupy diverse niches based on body mass, digestive system, social structure, anatomy and physiology (Bailey et al., 1996). Such characteristics determine which prey species are available to which predator species and therefore how much predation risk is experienced by prey in relation to different predator species. African lions (*Panthera leo*), the largest African predators, hunt in prides, ambush their prey and are generally unselective in their diet (Owen‐Smith & Mills, 2008a). In contrast, African wild dogs (*Lycaon pictus*; hereafter wild dogs) are cursorial predators that tend to concentrate on smaller prey species than lions (Owen‐Smith, 2015), although the large pack size of wild dogs can enable them to tackle larger prey (Creel, 1997; Radloff & Du Toit, 2004). Lions tend to be more active overnight whereas wild dogs are generally crepuscular, although temporal overlap in activity patterns can vary with environmental conditions (Rafiq et al., 2023). There is little recorded overlap between wild dog and lion prey species (31.6%; Hayward & Kerley, 2008), but smaller species such as impala (*Aepyceros melampus*; mean adult weight 50 kg) are vulnerable to both (Radloff & Du Toit, 2004). Medium‐bodied species such as blue wildebeest (mean adult weight 220 kg) are within the prey range of wild dogs but are less preferentially hunted, and larger species such as plains zebra (mean adult weight 310 kg) are primarily predated on by lions (Owen‐Smith & Mills, 2008b). Tsessebes (*Damaliscus lunatus*; mean adult weight 127 kg; Radloff & Du Toit, 2004) are infrequently recorded as prey by any predators (Hayward et al., 2006), possibly because tsessebes are fast and highly manoeuvrable (Wilson, unpublished data). Using GPS‐enabled collars deployed onto lions, wild dogs, impala, tsessebes, wildebeest and zebra, we explored variations in prey responses to predation risk from large‐bodied ambush (lion) and smaller‐bodied cursorial (wild dog) predators. Specifically, we tested the hypotheses that (i) prey decreased their movements during periods of high predator activity; (ii) predators selected different habitat types during periods of high and low activity; (iii) prey varied their habitat selection patterns based on predator activity‐dependent habitat selection; (iv) at a broader scale, prey utilization of areas used more intensively by active predators was lower than areas used less intensively and by inactive predatory regardless of habitat; (v) all prey responses (movement, habitat selection and utilization) were more closely correlated with lion than wild dog predation risk, given the larger size and ambush hunting technique of the former; (vi) responses to predation risk decreased with body mass for most herbivore species except for tsessebes, which experience lower predation pressure than other species; and (vii) prey responses were less extreme in the dry than rainy season, when forage availability and temperature were most constraining.

## 2 MATERIALS AND METHODS

### 2.1 Study area

**FIGURE 1:**
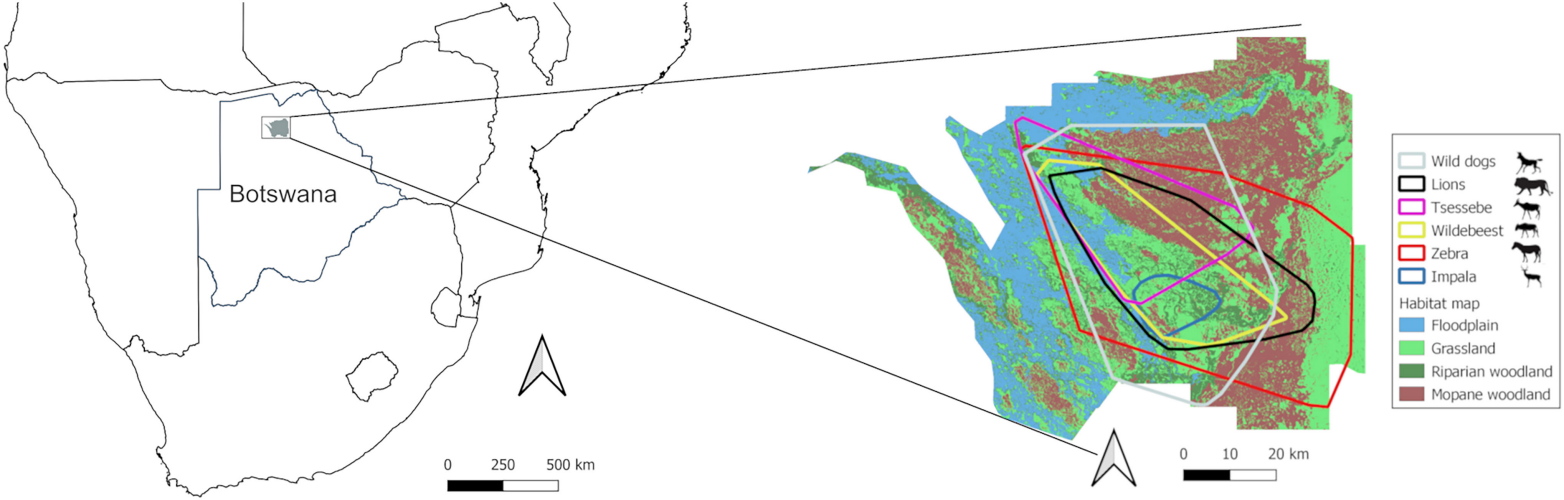
Map of study area depicting overlapping home ranges of all collared species. Data were collected in the Okavango Delta, Botswana, between 2014 and 2016.

The study was conducted in the south‐eastern part of the Okavango Delta, Botswana, including the Moremi Game Reserve and wildlife management areas NG33 and NG34. Habitats in the study area were simplified from Bennitt et al. (2014) into four types: floodplain, characterized by seasonal flooding and dominated by flood‐resistant grasses and sedges; grassland, characterized by open areas dominated by various grass species; mopane woodland, dominated by *Colophospermum mophane*; and mixed woodland, characterized by relatively high canopy cover and various woody species (Figure 1). We used seasonal rainfall patterns in the study area to define rainy (November–April) and dry (May–October) seasons. In 2014/2015, rainfall was lower (306 mm) than in 2015/2016 (430 mm), and the 2015 maximum flood extent (8884 km^2^) was higher than that of 2016 (7276 km^2^; Bennitt et al., 2019). The study area was inhabited by five large carnivore species (lion, wild dog, leopard *Panthera pardus*, spotted hyaena *Crocuta crocuta* and cheetah *Acinonyx jubatus*), and a large variety of herbivore species (including African elephant *Loxodonta africana*, giraffe *Giraffa camelopardalis*, hippo *Hippopotamus amphibius*, Cape buffalo *Syncerus caffer caffer*, plains zebra, blue wildebeest, greater kudu *Tragelaphus strepsiceros*, tsessebes and impala).

### 2.2 Collars

Four wild dogs, 6 lions, 5 impala, 8 tsessebes, 8 wildebeest and 14 zebra were collared over a 2‐year period between the beginning of the 2014 rainy season and the end of the 2016 dry season (Table S1). Although all herbivore collars included a drop‐off unit (Sirtrack, Hawkes Bay, New Zealand), three tsessebes, three wildebeest and one impala had to be re‐darted following drop‐off failure. All collared tsessebes, wildebeest and zebra were females, as they were representative of breeding herds and males could have damaged collars through fighting. All collared impala were harem‐holding males that represented breeding herds and were only collared during the dry season, when fights are uncommon. Both sexes of wild dogs and lions were collared. Each collared animal was adult and represented a distinct group, although occasionally some individuals would join up for short periods. Although not all carnivore home ranges overlapped with all herbivore home ranges, all collared individuals occupied similar areas with comparable habitat types (Figure 1).

All animals were darted by an experienced, qualified veterinarian registered in Botswana following the issuing of research permit EWT 8/36/4 XXIV (199) and associated darting permits by the Department of Wildlife and National Parks of Botswana. Two wildebeest were darted from a helicopter; all other animals were darted from a vehicle. Methods were approved by the Ethics and Welfare Committee of the Royal Veterinary College, London (URN 2013 1233). Wild dogs were immobilized with 50 mg ketamine, 55 mg xylazine and 1.1–1.2 mg atropine. Lions were immobilized with 60 mg ketamine, 25 mg tiletamine hydrochloride (as 50 mg zoletil, Virbac), 2 mg butorphanol tartrate and 6 mg medetomidine. All carnivores were reversed with atipamezole 60 min after darting. Impala were immobilized with 1.5 mg thiafentanil oxalate, 2 mg butorphanol tartrate and 1700 i.u. hyalase. Tsessebes were immobilized with 4 mg thiafentanil, 80 mg azaperone, 1700 i.u. hyalase and topped up post‐capture with 100 mg ketamine. Wildebeest were immobilized with 5 mg etorphine, 80 mg azaperone and 1700 i.u. hyalase. Zebra were immobilized with 7 mg etorphine, 80 mg azaperone and 1700 i.u. hyalase. All herbivores were reversed with naltrexone as soon as collars had been fitted. Dart sites were injected with local antibiotics to prevent infection. Heavily pregnant animals and those with small young were avoided and all individuals were observed rejoining their original groups. Hourly GPS fixes downloaded from the collars were used for all analyses. Collars were manufactured by the Royal Veterinary College, University of London; details of collar design can be found in Wilson et al. (2013).

### 2.3 Activity levels

All data manipulations and analyses were carried out in R v. 4.2.0 (R Core Team, 2022). We calculated seasonal hourly distances moved for all species using the adehabitatLT package (Calenge et al., 2009) and used them to define activity levels as high (hourly distance moved > mean hourly distance moved) or low (hourly distance moved < mean hourly distance moved). Distance data distribution was skewed to the right because small movements were more common than large ones. To determine whether carnivore activity level affected hourly distance moved by herbivores, we logged the response variable of hourly distance moved, then ran linear mixed models using the lme4 package (Bates, 2010) for each herbivore species with carnivore species, activity level, season and their interaction as the fixed effects; and the random effects of herbivore individual. Impala data were only available for the dry season, so season was not included in that model. Here and throughout, we used Akaike's information criterion corrected for small sample sizes (AIC_c_; Akaike, 1974) to identify the most parsimonious models. Goodness of fit was assessed using Kolmogorov–Smirnov (KS) tests for distribution, dispersion and outlier tests in the ‘DHARMa’ package (Hartig & Hartig, 2017).

### 2.4 Habitat selection

We split all datasets according to seasons and carnivore activity levels and generated utilization distributions (UDs) for each resulting subset using the kernel density method with the ‘href’ smoothing parameter in adehabitatHR (Calenge, 2006), which does not consider trajectories between points so was appropriate for datasets split into time chunks. We produced 95% kernel outlines and calculated the proportion of each habitat within them as a measure of availability. We used the utilization intensity from the UD to produce a weighted measure of use for each habitat, and compared use with availability to produce third‐order Manly selection ratios (Manly et al., 2002) using the adehabitatHS package (Calenge, 2007). We compared habitat selection between periods of high and low activity for wild dogs and lions by running multivariate analyses of variance (MANOVAs) with the selection ratios as the dependent variables and the fixed effects of season, activity level and their interaction as the predictor variables. For each herbivore–carnivore dyad, we ran MANOVAs with the selection ratios as the dependent variables and the fixed effects of species, predator activity level and their interaction as the predictor variables to compare selection ratios between predators and prey. We used stepwise model selection to identify the best‐fitting models.

### 2.5 Resource selection functions

We split GPS data from prey species into seasons and predator activity levels, then generated minimum convex polygons (MCPs) around each resulting dataset of GPS coordinates recorded by collars. We used the ‘sp’ package to generate an equal number of random points to the number of actual GPS points for each dataset, bounded within the respective MCPs. A 1:1 ratio was appropriate as the sample size was greater than 10,000 points (Northrup et al., 2013). Actual points were coded as ‘1’ and considered to have been used; random points were coded as ‘0’ and considered to have been available. For each used and available point, we extracted the level of utilization intensity (UI) from previously generated predator UDs, which represented the intensity at which a given pixel within a UD was used by a predator and therefore correlated with the likelihood of encountering a predator. We ran resource selection functions to determine whether prey species showed any avoidance of areas used more intensively by wild dogs and lions, particularly when predators were active. We ran generalized linear mixed models with a binomial distribution for each prey species in the dry and rainy seasons, with used/available as the response variable, a three‐way interaction among UI (scaled by subtracting the mean and dividing by the standard deviation), activity level and predator species as the fixed effects and individual prey as the random effect.

## 3 RESULTS

### 3.1 Collared animals

Impala were only collared during the dry season. Collar failures meant that some individuals of other species only contributed data during one season (Table S1). Not all individuals were collared over the same period. Only data from individuals collared for full seasons were used.

### 3.2 Activity patterns

**FIGURE 2:**
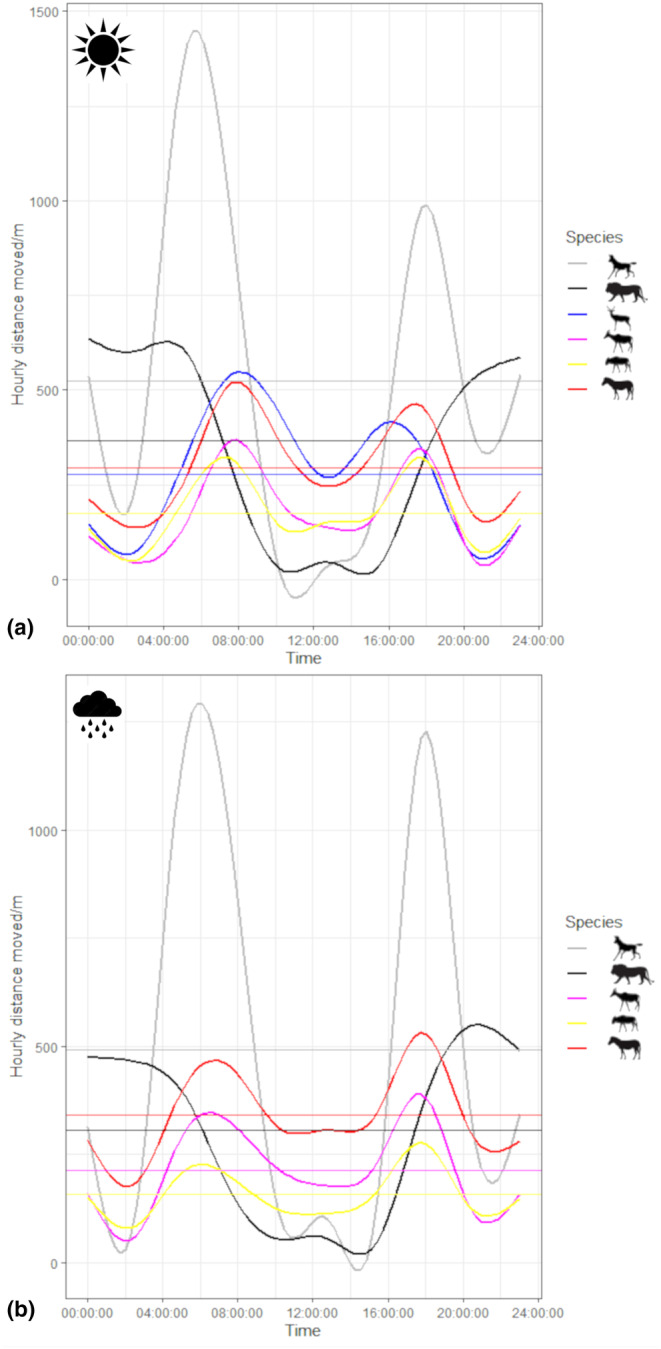
Circadian activity patterns (based on hourly distances) in the (a) dry and (b) rainy seasons for African wild dogs, lions, impala, tsessebes, zebra and wildebeest in the Okavango Delta, Botswana, between 2014 and 2016. Horizontal lines represent mean hourly distances for each species.

Wild dogs, tsessebes, wildebeest and zebra all had short periods of high activity in the early morning and late afternoon; lions were active from late afternoon to early morning and impala were active from early morning to late afternoon with a very short period of low activity in the middle of the day (Figure 2). Wild dogs showed high consistency in activity patterns across seasons, whereas other species showed some variation in activity period durations (Figure 2). Overall, zebra hourly distances were greatest, and tsessebes and wildebeest covered the least ground, regardless of activity. Impala, together with zebra, covered the highest distances during activity peaks and impala hourly distances dropped to match those of tsessebes and wildebeest during activity troughs, indicating that impala demonstrated the greatest amount of variation in hourly distance during periods of high and low activity (Figure 2).

**FIGURE 3:**
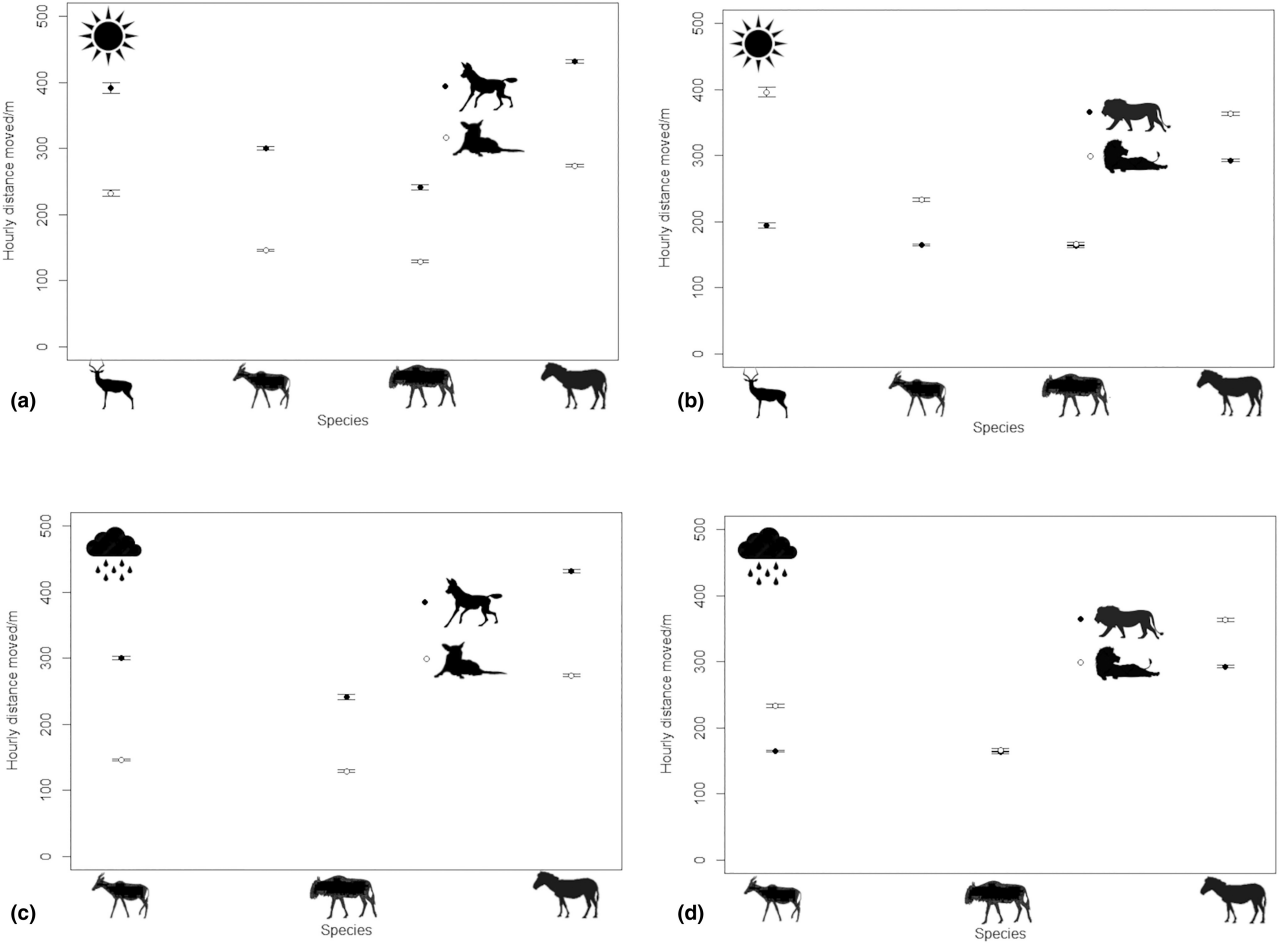
Hourly distances moved by impala, tsessebes, wildebeest and zebra during periods of high and low activity by (a) wild dogs and (b) lions during the dry season, and by (c) wild dogs and (d) lions during the rainy season. Black and white dots represent high and low levels of carnivore activity respectively. Data were collected between 2014 and 2016 in the Okavango Delta, Botswana.

For impala, the model with the interaction between carnivore species and activity level was the most parsimonious (AIC_c_ = 116,612, AIC_ω_ = 1.00, KS *p* = 0, dispersion *p* = .91 and outlier *p* = 0; Table S2); no other model was competitive. Models with the three‐way interaction among carnivore species, activity and season were the most parsimonious for tsessebes (AIC_c_ = 426,841, AIC_ω_ = 1.00, KS *p* = 0, dispersion *p* = .34, outlier *p* = 0; Table S2), wildebeest (AIC_c_ = 196,764, AIC_ω_ = 1.00, KS *p* = 0, dispersion *p* = .83, outlier *p* = 0; Table S2) and zebra (AIC_c_ = 573,219, AIC_ω_ = 1.00, KS *p* = 0, dispersion *p* = .39, outlier *p* = 0; Table S2); no other models were competitive. All herbivore species moved more and less when wild dogs showed high and low activity, respectively (Figure 3a,c). Most herbivores moved less and more when lions showed high and low activity, respectively (Figure 3), although wildebeest moved the same amount when lions showed high and low activity (Figure 3b,d). Differences between hourly herbivore distances moved during periods of high and low lion activity levels in the dry season were greatest for impala (Figure 3d) and smallest for wildebeest (Figure 3d). Although season was a factor in the most parsimonious models for tsessebes, wildebeest and zebra, there were no discernible patterns relating to seasonal variation in hourly distances (Figure 3). Overall, differences between hourly distances had a stronger positive relationship with wild dog activity and a weaker negative relationship with lion activity.

### 3.3 Habitat selection

Wild dogs and lions differed in their habitat selection ratios between seasons (wild dogs:$F_{15}^{4}$ = 3.705, *p* = .038; lions: $F_{22}^{4}$ = 3.178, *p* = .037) but not between activity levels (wild dogs:$F_{14}^{4}$ = 0.095, *p* = .982; lions: $F_{23}^{4}$ = 0.086, *p* = .986). Habitat selection ratios differed between prey and predators during most seasons but were not affected by predator activity level (Table 1).

**TABLE 1:** Comparison of habitat selection between prey and predator dyads during the dry and rainy seasons and periods of high and low predator activity.

Prey	Predator	Season	Effect of predator activity level	Difference in habitat selection between prey and predators	Habitats avoided more by prey than predators	Habitats selected more by prey than predators
Impala	Wild dog	Dry	$F_{12}^{4}$ = 0.146, *p* = .961	$F_{13}^{4}$ **= 18.727, *p* < .001**	**Floodplain (*F***_**1**_ **= 9.317, *p* = .008)** **Mopane (*F***_**1**_ **= 23.183, *p* < .001)**	**Woodland (*F***_**1**_ **= 4.967, *p* = .041)**
Impala	Lion	Dry	$F_{12}^{4}$ = 0.907, *p* = .483	$F_{13}^{4}$ **= 31.235, *p* < .001**	**Floodplain (*F***_**1**_ **= 44.945, *p* < .001)**	
Tsessebe	Wild dog	Dry	$F_{14}^{4}$ = 0.042, *p* = .996	$F_{15}^{4}$ **= 6.010, *p* = .004**		**Floodplain (*F***_**1**_ **= 11.843, *p* = .003)**
Tsessebe	Wild dog	Rainy	$F_{16}^{4}$ = 0.062, *p* = .992	$F_{17}^{4}$ **= 3.905, *p* = .020**		**Grassland (*F***_**1**_ **= 3.730, *p* = .068)**
Tsessebe	Lion	Dry	$F_{18}^{4}$ = 0.611, *p* = .660	$F_{19}^{4}$ **= 5.770, *p* = .003**	**Woodland (*F***_**1**_ **= 8.822, *p* = .007)**	
Tsessebe	Lion	Rainy	$F_{20}^{4}$ = 0.016, *p* = .987	$F_{21}^{4}$ **= 6.042, *p* = .002**	**Floodplain (*F***_**1**_ **= 8.071, *p* = .009)**	**Grassland (*F***_**1**_ **= 5.363, *p* = .029)**
Wildebeest	Wild dog	Dry	$F_{12}^{4}$ = 0.024, *p* = .999	$F_{13}^{4}$ **= 9.694, *p* < .001**	**Mopane (*F***_**1**_ **= 13.218, *p* = .002)**	**Floodplain (*F***_**1**_ **= 9.263, *p* = .008)**
Wildebeest	Wild dog	Rainy	$F_{16}^{4}$ = 0.020, *p* = .999	$F_{17}^{4}$ **= 3.014, *p* = .048**	**Mopane (*F***_**1**_ **= 10.146, *p* = .005)**	
Wildebeest	Lion	Dry	$F_{16}^{4}$ = 0.672, *p* = .621	$F_{17}^{4}$ = 1.713, *p* = .194		
Wildebeest	Lion	Rainy	$F_{20}^{4}$ = 0.304, *p* = .872	$F_{21}^{4}$ **= 3.083, *p* = .038**	**Floodplain (*F***_**1**_ **= 5.263, *p* = .031)**	
Zebra	Wild dog	Dry	$F_{18}^{4}$ = 0.012, *p* = .999	$F_{19}^{4}$ **= 8.152, *p* < .001**	**Mopane (*F***_**1**_ **= 18.201, *p* < .001)**	**Floodplain (*F***_**1**_ **= 8.470, *p* = .008)** **Woodland (F**_**1**_ **= 11.163, *p* = .003)**
Zebra	Wild dog	Rainy	$F_{24}^{4}$ = 0.023, *p* = .999	$F_{25}^{4}$ = 1.350, *p* = .279		
Zebra	Lion	Dry	$F_{22}^{4}$ = 1.158, *p* = .356	$F_{23}^{4}$ = 2.448, *p* = .075		Mopane (*F*_1_ = 7.720, *p* = .01)
Zebra	Lion	Rainy	$F_{29}^{4}$ = 0.128, *p* = .971	$F_{30}^{4}$ **= 8.921, *p* < .001**	**Floodplain (*F***_**1**_ **= 5.624, *p* = .024)**	**Mopane (*F***_**1**_ **= 34.488, *p* < .001)**

*Note*: Data were collected between 2014 and 2016 in the Okavango Delta, Botswana. Bold text indicates significant results.

**FIGURE 4:**
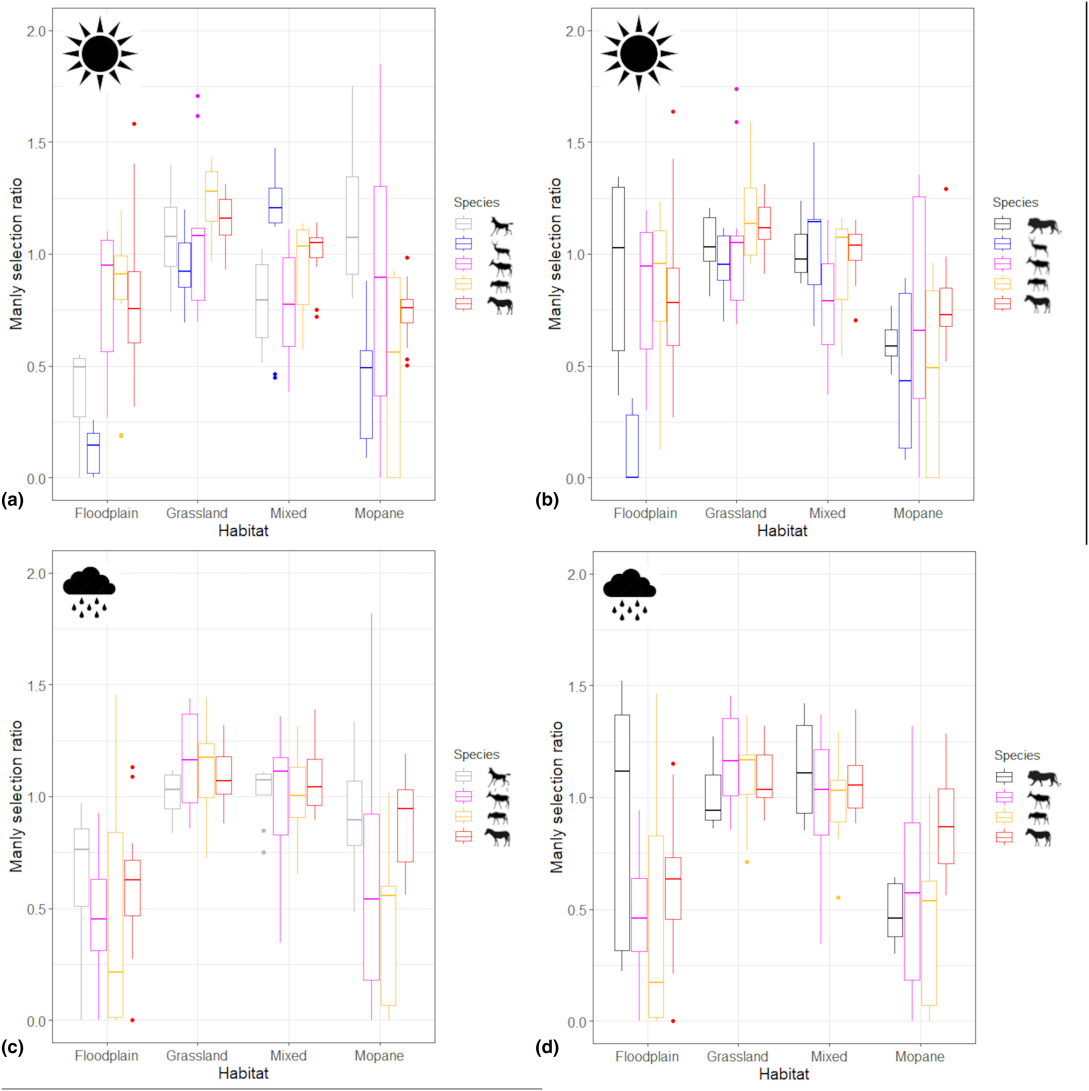
Habitat selection ratios for impala, tsessebes, wildebeest and zebra in comparison to African wild dogs in the dry (a) and rainy (c) seasons, and to lions in the (b) dry and (d) rainy seasons. Data were collected between 2014 and 2016 in the Okavango Delta, Botswana.

During the dry season, all species other than wild dogs and tsessebes avoided mopane woodland; wild dogs, impala and zebra avoided floodplains; wildebeest and zebra selected grassland; and selection or avoidance for mixed woodland varied with species (Figure 4). During the rainy season, all species other than lions avoided floodplains and lions, tsessebes and wildebeest avoided mopane woodland (Figure 4). Impala appeared to show stronger selection patterns than other species and, in general, habitat selection was stronger during the dry than rainy season (Figure 4).

### 3.4 Resource selection functions

**FIGURE 5:**
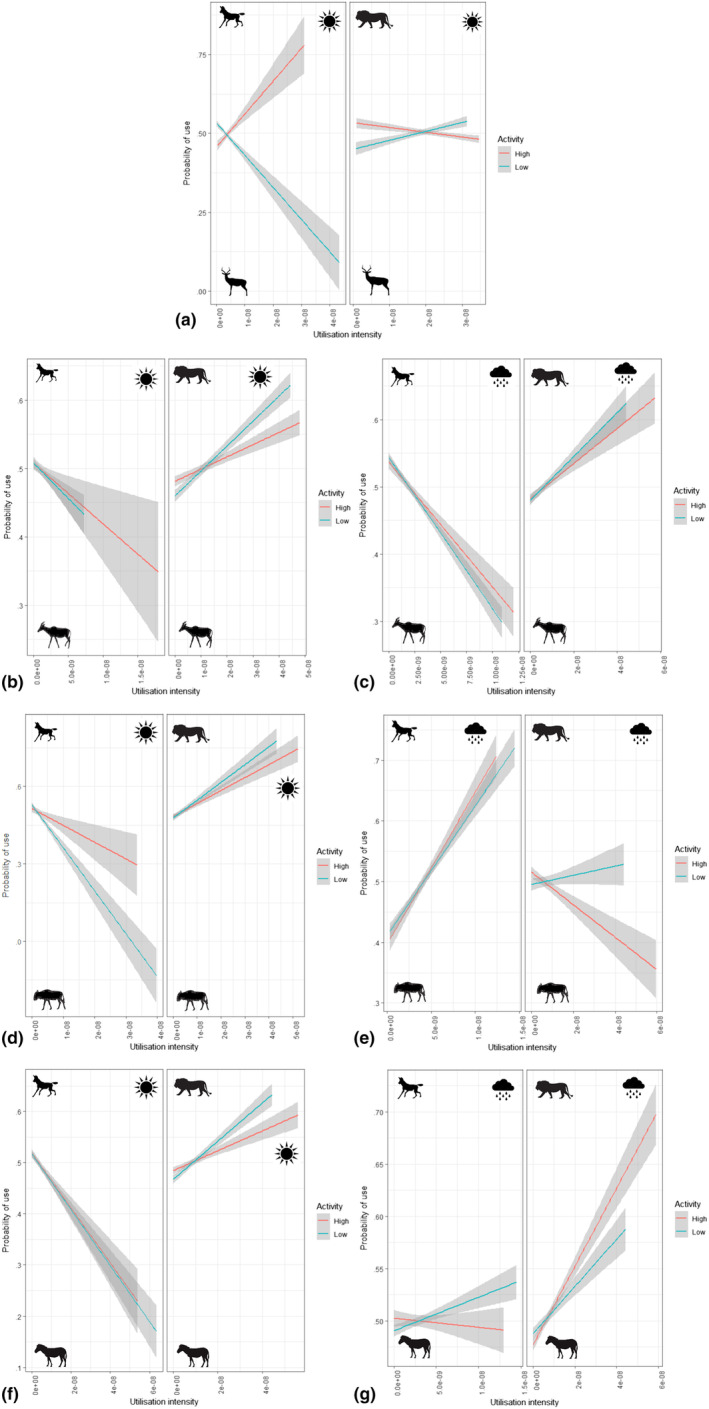
Probability of use of locations in relation to utilization intensity by wild dogs and lions for (a) impala, tsessebes in the (b) dry and (c) rainy seasons, wildebeest in the (d) dry and (e) rainy seasons and zebra in the (f) dry and (g) rainy seasons. Data were collected in the Okavango Delta, Botswana, in 2014–2016.

For impala, the most parsimonious model included the three‐way interaction among UI, activity level and predator species (AIC_c_ = 79,230, AIC_ω_ = 1.00, KS *p* = .35, dispersion *p* = .90 and outlier *p* = .09; Table S3; no other models were competitive). Impala were more likely to use areas favoured by active dogs and less likely to use areas with high dog UI during periods of low activity (Figure 5a). The opposite was true for lions, but the effect was weaker (Figure 5a). For tsessebes during the dry season, the most parsimonious model included the two‐way interactions between UI and activity, UI and predator species, and activity and predator species (AIC_c_ = 129,604, AIC_ω_ = 0.57, KS *p* = .31, dispersion *p* = .64, outlier *p* = .44; Table S3); the model that included the three‐way interaction among UI, activity and predator species was also competitive (ΔAIC = 1.04, AIC_ω_ = 0.34, KS *p* = .14, dispersion *p* = .06 and outlier *p* = .19; Table S3) but parameter estimates were similar so we did not use model averaging (Cade, 2015). Tsessebes were less likely to use areas favoured by wild dogs, with little effect of activity (Figure 5b); the opposite was true for lions, and tsessebes were more likely to occupy areas with high lion use during low activity periods (Figure 5b). For tsessebes during the rainy season, the most parsimonious model included the interaction between UI and predator species (AIC_c_ = 155,867, AIC_ω_ = 0.48, KS *p* = .35, dispersion *p* = .64 and outlier *p* = .48; Table S3); the model that also included the fixed effect of activity level was competitive (ΔAIC = 1.95, AIC_ω_ = 0.18, KS *p* = .38, dispersion *p* = .63, outlier *p* = .55; Table S3) but parameter estimates were similar so we did not use model averaging (Cade, 2015). Tsessebes were less likely to use areas favoured by wild dogs and more likely to use those with higher lion UI (Figure 5c); slight differences indicated that these effects were more extreme for periods of low activity in both predator species.

For wildebeest in the dry season, the most parsimonious model included the three‐way interaction among UI, activity level and predator species (AIC_c_ = 53,729, AIC_ω_ = 1.00; KS *p* = .52, dispersion *p* = .28 and outlier *p* = .61; Table S3); no other models were competitive. Wildebeest were less likely to use areas with high wild dog UI and more likely to use those favoured by lions; for both predator species, this effect was more pronounced for areas used during periods of low activity (Figure 5d). For wildebeest in the rainy season, the most parsimonious model included the three‐way interaction among UI, activity level and predator species (AIC_c_ = 84,325, AIC_ω_ = 0.73; KS *p* = .04, dispersion *p* = .01 and outlier *p* = .48; Table S3); no other models were competitive. Wildebeest were more likely to use areas favoured by wild dogs regardless of activity level, less likely to use areas occupied by lions during active periods and showed little indication of preference or avoidance of areas used by lions during periods of low activity (Figure 5e).

For zebra in the dry season, the most parsimonious model included the three‐way interaction among UI, activity level and predator species (AIC_c_ = 145,730, AIC_ω_ = 0.62; KS *p* = .49, dispersion *p* = .84, outlier *p* = .41; Table S3); no other models were competitive. Zebra were less likely to use areas occupied by wild dogs and more likely to use those favoured by lions; activity had relatively little effect on wild dogs, but zebra were more likely to be encountered in areas used more by lions during period of low activity (Figure 5f). For zebra during the rainy season, the most parsimonious model included the three‐way interaction among UI, activity level and predator species (AIC_c_ = 259,276, AIC_ω_ = 0.99; KS *p* = .19, dispersion *p* = .02 and outlier *p* = .94; Table S3); no other models were competitive. Zebra were more likely to use areas occupied by wild dogs during periods of low activity, and much more likely to use areas favoured by lions, particularly during periods of high lion activity (Figure 5g).

## 4 DISCUSSION

All herbivore species display adaptations to avoid predation in terms of anatomy and physiology (Wilson et al., 2018); proactive predator avoidance behaviours can also be vital in ensuring herbivore survival (Creel et al., 2005). This extensive study of multiple behavioural adaptations in four herbivore species demonstrates how they may minimise predation risk based on predator spatio‐temporal activity patterns. Except for wildebeest, herbivores covered larger distances during periods of higher wild dog than lion activity. Predator activity level had no effect on carnivore or herbivore habitat selection patterns, but carnivores and herbivores differed overall in some habitat selection trends. The likelihood of wild dogs using an area had relatively little effect on the likelihood of most herbivore species using an area, although impala were much more likely to be in areas used by wild dogs during periods of high than low predator activity, most likely because wild dogs were seeking impala rather than the other way around. Indeed, the preference among predators for particular areas is likely to be driven primarily by seasonal prey movements at a large scale and prey ‘catchability’ at a finer scale (Hopcraft et al., 2005), so spatial and temporal activity patterns of carnivores and herbivores are likely to be correlated. Apart from zebra during the rainy season, most herbivores were more likely to use areas favoured by lions during periods of low than high lion activity. Temporal variation in lion activity level was more strongly correlated with herbivore movement patterns than wild dog activity level, but herbivores showed less persistent responses to spatial variation in activity by either predator. High predator activity levels could be linked to behaviour other than hunting, such as patrolling, but Dröge et al. (2017) provided evidence of higher kill rates during these periods, supporting the concept that high predator activity is linked to higher predation risk. Seasonal variation in habitat selection suggested that herbivores were more driven by resource availability than predation risk avoidance during the limiting dry season.

Data from different species were not collected entirely simultaneously, although there was substantial overlap among all species (Table S1); only impala were collared outside of the collaring period for predators. There were some differences in rainfall and flood levels between years that could have affected results to some extent. However, circadian activity patterns from these data broadly match those from other studies (Cozzi et al., 2012; Rafiq et al., 2023; Veldhuis et al., 2020) and habitat selection patterns are unlikely to have changed substantially between consecutive years. Although predator group sizes and territories, which could affect the intensity of use of an area by predators, can change over time, the relatively short time span (2 years) of the study reduces that risk. Goodness of fit varied with models, being lower for the hourly distance analyses than the RSF analyses. Hourly distance moved is likely to be affected by variables that were not considered, such as ambient temperature, but we were not able to capture all relevant data. Most RSF models fit well, although wildebeest and zebra rainy season models had issues with the distribution and dispersion measures. Results from models with low goodness of fit should be interpreted with caution, although the inclusion of random effects can affect the residuals (Hartig & Hartig, 2017).

In accordance with existing literature (Dröge et al., 2017; Hayward & Slotow, 2009), wild dogs and lions showed contrasting temporal patterns of activity, with the former primarily active during crepuscular periods, and the latter being mainly nocturnal. We did not extend the study to include the effect of moonlight, which has been shown to alter carnivore activity patterns (Cozzi et al., 2012). Wild dogs are vulnerable to injury and death from lions (Dröge et al., 2017), so avoiding their peak activity periods would reduce such risks in a similar manner to herbivores reducing predation risk. Given the contrasting activity patterns of lion and wild dogs, it would not be feasible for prey to avoid high activity periods for both carnivore species, but in accordance with our hypothesis, herbivores were covering less ground during periods of high than low lion activity; the opposite effect was detected for wild dog activity levels.

No animals were active during the middle of the day, most likely because of thermoregulation and the need to conserve energy and water during the heat of the day. Lower temperatures at night would promote nocturnal foraging, but the predation risk from lions is probably too high; nocturnal foraging develops in large herbivores in systems where lions are absent (Veldhuis et al., 2020). Herbivore behaviour and activity were more strongly correlated with the spatio‐temporal activity patterns of lions than wild dogs. It is likely that the difference in body mass and dietary breadth between lions and wild dogs (Makin et al., 2017) had more of an impact on temporal partitioning than the difference in their hunting strategies. Ambush hunters are more predictable than cursorial predators in terms of spatial distribution (Courbin et al., 2019), leading to stronger proactive avoidance by prey (Droge et al., 2017), but the two hunting strategies would not necessarily cause a difference in the predictability of temporal activity patterns.

Prey will increase their speed of movement when predators are nearby to reduce short‐term predation risk (Droge et al., 2019), but greater movement could raise the possibility of detection by predators when prey may not be aware of them. Impala showed the greatest difference in hourly distance moved between periods of high and low lion activity. There was no evidence that wildebeest hourly movements correlated with lion activity levels, providing some support for the effect of herbivore body mass, but this relationship did not hold for tsessebes, which may avoid predation by responding to spatio‐temporal variation in predation risk. Wildebeest are short‐grass specialists that may have lower availability of productive foraging patches than more generalist herbivores, and covering greater distances could cause them to leave a productive patch (Martin & Owen‐Smith, 2016), potentially affecting the balance between energetic intake and predation risk. Previous studies found that wildebeest reacted more strongly with vigilance to predation risk from wild dogs (Droge et al., 2017), despite being more commonly hunted by lions (Owen‐Smith, 2015) and that they showed little reaction to lion roars (Dannock et al., 2019), so they appear to be relatively insensitive to lion predation risk.

In contrast to previous studies (Courbin et al., 2019; Fischhoff et al., 2007), we found no evidence that carnivores selected different habitat types based on activity level. Cursorial predators are considered to be habitat generalists, seeking prey through opportunistic encounters (Donadio & Buskirk, 2016), whereas ambush predators are thought to target prey‐rich areas at a broad scale and locations where their chances of capturing prey are greatest at a finer scale (Hopcraft et al., 2005), making their habitat selection patterns more predictable than those of their cursorial counterparts (Makin et al., 2017). Prey species are thought to avoid risky habitats during risky times (Palmer et al., 2017), but our study found no effect of predator activity level on habitat selection for any herbivore species. Most herbivore species differed in their overall habitat selection patterns to predators, except for wildebeest and zebra, which selected similar habitats to lions during the dry season, which may have been linked to forage and water scarcity that would have dictated herbivore distribution to a greater extent than predation risk (Lone et al., 2017; Palmer et al., 2017). This supported our hypothesis that predation risk avoidance behaviour would be less extreme during the resource‐limited dry season. Zebra also selected habitats to the same extent as wild dogs during the rainy season, but zebra are rarely preyed upon by wild dogs in southern Africa (Hayward et al., 2006). Impala avoided floodplains compared to both carnivore species, but floodplains probably did not offer productive foraging conditions to these mixed feeders during the dry season when they may have turned to browsing, which is not available on floodplains. Tsessebes, wildebeest and zebra all avoided floodplains to a greater extent than lions during the rainy season, supporting our hypothesis that anti‐predation behaviour would be more evident during that period. However, floodplains offer less productive forage than other habitats during the rainy season (Bennitt et al., 2014), so forage availability could also have influenced herbivore distribution.

Overall, the habitat selection patterns of all herbivore species seemed to be more driven by forage availability than predator avoidance and did not appear to be affected by herbivore body mass or species‐specific predation risk. Resource distribution varies over a much longer timescale than direct predation risk (Thaker et al., 2011), and the difference in prioritization of resource acquisition compared to predation risk reduction is likely to depend on the abundance of the former and the density of the latter (Tolon et al., 2009), so could fluctuate seasonally and annually. Not all study individuals were collared at the same time, and rainfall and flood extent were higher in the second year of data collection so there could have been environmental differences between seasons in different years or in densities or predators or prey that could have affected the results, but the data were collected within a relatively short time in the same geographical area. Ideally, predator and prey data would have been collected simultaneously, but the financial and logistical resources required by such as study are prohibitive. The results presented here were gathered from multiple species of predator and prey collared in the same system over 2 years, which represents an unusual dataset that can yield valuable insights, even with the caveat of incomplete temporal synchrony. This study focused on proactive behaviours to reduce long‐term predation risk, so more investigation is required to determine whether individuals responded to short‐term predation threats linked to carnivore presence by changing habitats, as has been observed elsewhere (Valeix et al., 2009).

Herbivore species differed in their likelihood to use areas favoured by wild dogs and lions, and these patterns sometimes, but not always, varied with predator activity level; such variation was more common in relation to space use by lions than wild dogs. Generally, herbivores were less likely to use areas with high wild dog use and more likely to use those favoured by lions. In most cases, where activity level was correlated with herbivore behaviour, herbivores were more likely to use areas with high lion use during periods of low lion activity, although the opposite was true for zebra during the rainy season; perhaps lions were actively seeking zebra during that period. Our results support previous findings that prey should use risky places less at risky times (Courbin et al., 2019; Donadio & Buskirk, 2016; Gehr et al., 2018), even without a direct interaction with a predator. Predator–prey interactions are dynamic, with predators often seeking areas of high prey abundance and catchability (Hopcraft et al., 2005; Laundre, 2010), so these results most likely stem from predators selecting areas where prey are more likely to be encountered (Creel, 2018). All the prey species considered here are highly mobile, and would generally use flight to escape predation rather than defence, so their ability to flee balances to a certain extent the risk of encountering predators, in contrast to species who would stand their ground and defend against an attack (Wikenros et al., 2015). Our results suggest that the likelihood of a predator using a given area, as extracted from utilization distributions, is a more effective predictor of herbivore space use than habitat selection alone, perhaps because the Okavango Delta is composed of a very dense mosaic of different habitat types, often occurring in small patches (Bennitt et al., 2014).

Collecting detailed information from multiple species simultaneously can be difficult and costly, but such studies can provide valuable insight into adaptations allowing herbivores to consider predation risk and resource availability in daily decisions on foraging sites and movement patterns. Our results suggest that, for most prey species, minimizing movement during periods of high lion activity may be the best approach to manage predation risk, since the large body size of lions makes them the largest predation threat and their ambush tactics make them more predictable. Hunting predators will target locations with high herbivore encounter probability, which often coincides with limiting resources; herbivores avoiding predation risk are more likely to visit such areas when predators are less active. Regular activity patterns and selection of particular areas or resources by predators can make long‐term predation risk somewhat predictable by prey, which can employ mechanisms to minimize risk. However, it is rarely possible to predict the exact location of predators, so prey must also engage in reactive responses to direct threats caused by predator presence, and such behaviour should also be studied using high‐resolution GPS datasets from multiple predator and prey species.

## AUTHOR CONTRIBUTIONS

**Emily Bennitt:** Conceptualization (lead); data curation (equal); formal analysis (lead); writing – original draft (lead). **Hattie L. A. Bartlam‐Brooks:** Conceptualization (supporting); writing – review and editing (supporting). **Tatjana Y. Hubel:** Data curation (supporting); writing – review and editing (supporting). **Neil R. Jordan:** Data curation (supporting); project administration (supporting); writing – review and editing (supporting). **John W. McNutt:** Project administration (supporting); resources (supporting); writing – review and editing (supporting). **Alan M. Wilson:** Data curation (lead); funding acquisition (lead); project administration (lead); supervision (lead); writing – review and editing (supporting).

## Supporting information


Data S1

## Data Availability

Data are available through the Dryad Digital Repository, https://doi.org/10.5061/dryad.w0vt4b8zr.
