# Supplementary material for: Proactive cursorial and ambush predation risk avoidance in four African herbivore species
Source: Ecol Evol. 2024 Jun 5;14(6):e11529. doi: 10.1002/ece3.11529 (PMC11150757; doi:10.1002/ece3.11529)
Supplement: Supplementary file 1 — Data S1 [file ECE3-14-e11529-s001.zip › Supplementary information.docx]

Table S1. Collar-bearing periods for African wild dogs, lions, impala, tsessebe, wildebeest and zebra between 2014 – 2016 in the Okavango Delta, Botswana. Shading represents periods when animals were collared.

| ID | Species | Sex | 2014 | 2015 | | 2016 |
| --- | --- | --- | --- | --- | --- | --- |
|  |  |  | Rainy | Dry | Rainy | Dry |
| D1 | Wild dog | F |  |  |  |  |
| D2 | Wild dog | M |  |  |  |  |
| D3 | Wild dog | M |  |  |  |  |
| D4 | Wild dog | M |  |  |  |  |
| L1 | Lion | M |  |  |  |  |
| L2 | Lion | F |  |  |  |  |
| L3 | Lion | F |  |  |  |  |
| L4 | Lion | F |  |  |  |  |
| L5 | Lion | M |  |  |  |  |
| L6 | Lion | F |  |  |  |  |
| I1 | Impala | M |  |  |  |  |
| I2 | Impala | M |  |  |  |  |
| I3 | Impala | M |  |  |  |  |
| I4 | Impala | M |  |  |  |  |
| I5 | Impala | M |  |  |  |  |
| T1 | Tsessebe | F |  |  |  |  |
| T2 | Tsessebe | F |  |  |  |  |
| T3 | Tsessebe | F |  |  |  |  |
| T4 | Tsessebe | F |  |  |  |  |
| T5 | Tsessebe | F |  |  |  |  |
| T6 | Tsessebe | F |  |  |  |  |
| T7 | Tsessebe | F |  |  |  |  |
| T8 | Tsessebe | F |  |  |  |  |
| W1 | Wildebeest | F |  |  |  |  |
| W2 | Wildebeest | F |  |  |  |  |
| W3 | Wildebeest | F |  |  |  |  |
| W4 | Wildebeest | F |  |  |  |  |
| W5 | Wildebeest | F |  |  |  |  |
| W6 | Wildebeest | F |  |  |  |  |
| W7 | Wildebeest | F |  |  |  |  |
| W8 | Wildebeest | F |  |  |  |  |
| Z1 | Zebra | F |  |  |  |  |
| Z2 | Zebra | F |  |  |  |  |
| Z3 | Zebra | F |  |  |  |  |
| Z4 | Zebra | F |  |  |  |  |
| Z5 | Zebra | F |  |  |  |  |
| Z6 | Zebra | F |  |  |  |  |
| Z7 | Zebra | F |  |  |  |  |
| Z8 | Zebra | F |  |  |  |  |
| Z9 | Zebra | F |  |  |  |  |
| Z10 | Zebra | F |  |  |  |  |
| Z11 | Zebra | F |  |  |  |  |
| Z12 | Zebra | F |  |  |  |  |
| Z13 | Zebra | F |  |  |  |  |
| Z14 | Zebra | F |  |  |  |  |

Table S2. Most parsimonious LMM model outputs for hourly distance moved by prey (impala, tsessebe, wildebeest and zebra) in relation to predator (African wild dog and lion) activity level (high or low) and season (dry or rainy). Data were collected between 2014-2016 in the Okavango Delta, Botswana.

| Prey species | Predictor | Estimate | Standard error | t-value |
| --- | --- | --- | --- | --- |
| Impala | Predator (Lion) | -1.31 | 0.04 | -37.17 |
|  | Activity (Low) | -1.30 | 0.03 | -38.24 |
|  | Predator (Lion): Activity (Low) | 2.22 | 0.05 | 47.93 |
| Tsessebe | Predator (Lion) | -1.23 | 0.03 | -47.44 |
|  | Activity (Low) | -1.41 | 0.03 | -56.12 |
|  | Season (Rainy) | 0.05 | 0.03 | 1.64 |
|  | Predator (Lion): Activity (Low) | 1.97 | 0.03 | 57.42 |
|  | Predator (Lion): Season (Rainy) | 0.35 | 0.04 | 9.89 |
|  | Activity (Low): Season (Rainy) | 0.44 | 0.03 | 12.86 |
|  | Predator (Lion): Activity (Low): Season (Rainy) | -0.48 | 0.05 | -10.40 |
| Wildebeest | Predator (Lion) | -1.08 | 0.04 | -27.52 |
|  | Activity (Low) | -1.36 | 0.04 | -35.68 |
|  | Season (Rainy) | -0.11 | 0.04 | -2.64 |
|  | Predator (Lion): Activity (Low) | 1.65 | 0.05 | 31.68 |
|  | Predator (Lion): Season (Rainy) | 0.50 | 0.05 | 10.09 |
|  | Activity (Low): Season (Rainy) | 0.62 | 0.05 | 12.91 |
|  | Predator (Lion): Activity (Low): Season (Rainy) | -0.74 | 0.07 | -10.85 |
| Zebra | Predator (Lion) | -0.78 | 0.02 | -36.86 |
|  | Activity (Low) | -0.78 | 0.02 | -37.62 |
|  | Season (Rainy) | -0.03 | 0.02 | -1.37 |
|  | Predator (Lion): Activity (Low) | 1.34 | 0.03 | 47.78 |
|  | Predator (Lion): Season (Rainy) | 0.23 | 0.03 | 8.56 |
|  | Activity (Low): Season (Rainy) | 0.25 | 0.03 | 9.50 |
|  | Predator (Lion): Activity (Low): Season (Rainy) | 0.38 | 0.04 | -10.75 |

Table S3. Most parsimonious binomial GLMM model outputs for resource selection functions for prey (impala, tsessebe, wildebeest and zebra) in relation to predator (pred: African wild dog and lion) utilization intensity (UI) according to activity level (act: high or low). Data were split seasonally (dry and rainy) and collected between 2014-2016 in the Okavango Delta, Botswana.

| Prey species | Season | Predictor | Estimate | Standard error | z-value | P-value |
| --- | --- | --- | --- | --- | --- | --- |
| Impala | Dry | UI | 0.47 | 0.068 | 6.03 | <0.001 |
| AIC=79230 |  | Act(Low) | -0.69 | 0.07 | -9.60 | <0.001 |
| AICω=1.00 |  | Pred(Lion) | -0.27 | 0.06 | -4.31 | <0.001 |
|  |  | UI:Act(Low) | -0.94 | 0.09 | -10.05 | <0.001 |
|  |  | UI:Pred(Lion) | -0.54 | 0.08 | -6.76 | <0.001 |
|  |  | Act(Low):Pred(Lion) | 0.56 | 0.08 | 7.16 | <0.001 |
|  |  | UI:Act(Low):Pred(Lion) | 1.14 | 0.10 | 11.58 | <0.001 |
| Tsessebe | Dry | UI | -0.28 | 0.10 | -2.90 | 0.004 |
| AIC=129604 |  | Act(Low) | 0.03 | 0.02 | 1.55 | 0.122 |
| AICω=0.57 |  | Pred(Lion) | 0.09 | 0.05 | 1.69 | 0.092 |
|  |  | UI:Act(Low) | 0.07 | 0.02 | 4.94 | <0.001 |
|  |  | UI:Pred(Lion) | 0.38 | 0.09 | 4.06 | <0.001 |
|  |  | Act(Low):Pred(Lion) | -0.07 | 0.03 | -2.41 | 0.016 |
| Tsessebe | Dry | UI | -0.18 | 0.14 | -1.25 | 0.212 |
| ΔAIC=1.04 |  | Act(Low) | 0.04 | 0.08 | -0.52 | 0.606 |
| AICω=0.34 |  | Pred(Lion) | 0.04 | 0.07 | 0.55 | 0.580 |
|  |  | UI:Act(Low) | -0.08 | 0.16 | -0.50 | 0.616 |
|  |  | UI:Pred(Lion) | 0.27 | 0.14 | 1.96 | 0.050 |
|  |  | Act(Low):Pred(Lion) | -0.01 | 0.08 | -0.03 | 0.977 |
|  |  | UI:Act(Low):Pred(Lion) | 0.16 | 0.16 | 0.97 | 0.332 |
| Tsessebe | Rainy | UI | -0.57 | 0.03 | -20.01 | <0.001 |
| AIC=155867 |  | Pred(Lion) | 0.17 | 0.02 | 10.69 | <0.001 |
| AICω=0.48 |  | UI:Pred(Lion) | 0.65 | 0.03 | 22.47 | <0.001 |
| Tsessebe | Rainy | UI | -0.57 | 0.03 | -20.00 | <0.001 |
| ΔAIC=1.95 |  | Pred(Lion) | 0.17 | 0.02 | 10.60 | <0.001 |
| AICω=0.18 |  | Act(Low) | 0.01 | 0.01 | 0.23 | 0.82 |
|  |  | UI:Pred(Lion) | 0.65 | 0.03 | 22.46 | <0.001 |
| Wildebeest | Dry | UI | -0.17 | 0.05 | -3.04 | 0.002 |
|  |  | Act(Low) | -0.06 | 0.03 | -1.90 | 0.057 |
|  |  | Pred(Lion) | -0.01 | 0.03 | 0.19 | 0.852 |
|  |  | UI:Act(Low) | -.033 | 0.07 | -4.74 | <0.001 |
|  |  | UI:Pred(Lion) | 0.31 | 0.06 | 5.62 | <0.001 |
|  |  | Act(Low):Pred(Lion) | 0.06 | 0.04 | 1.39 | 0.164 |
|  |  | UI:Act(Low):Pred(Lion) | 0.39 | 0.07 | 5.28 | <0.001 |
| Wildebeest | Rainy | UI | 0.97 | 0.06 | 15.47 | <0.001 |
|  |  | Act(Low) | -0.02 | 0.03 | -0.67 | 0.504 |
|  |  | Pred(Lion) | -0.16 | 0.03 | -5.67 | <0.001 |
|  |  | UI:Act(Low) | -0.08 | 0.07 | -1.09 | 0.276 |
|  |  | UI:Pred(Lion) | -0.98 | 0.06 | -15.83 | <0.001 |
|  |  | Act(Low):Pred(Lion) | -0.01 | 0.04 | -0.05 | 0.959 |
|  |  | UI:Act(Low):Pred(Lion) | 0.18 | 0.07 | 2.47 | 0.013 |
| Zebra | Dry | UI | -0.20 | 0.02 | -8.27 | <0.001 |
|  |  | Act(Low) | -0.01 | 0.02 | -0.50 | 0.617 |
|  |  | Pred(Lion) | 0.03 | 0.02 | 1.61 | 0.107 |
|  |  | UI:Act(Low) | -0.01 | 0.03 | -0.19 | 0.850 |
|  |  | UI:Pred(Lion) | 0.27 | 0.03 | 10.47 | <0.001 |
|  |  | Act(Low):Pred(Lion) | -0.02 | 0.03 | -0.64 | 0.524 |
|  |  | UI:Act(Low):Pred(Lion) | 0.07 | 0.03 | 2.21 | 0.027 |
| Zebra | Rainy | UI | 0.03 | 0.03 | 0.99 | 0.322 |
|  |  | Act(Low) | 0.02 | 0.02 | 1.35 | 0.178 |
|  |  | Pred(Lion) | -0.04 | 0.02 | -2.22 | 0.026 |
|  |  | UI:Act(Low) | 0.10 | 0.03 | 3.13 | 0.002 |
|  |  | UI:Pred(Lion) | 0.08 | 0.03 | 2.88 | 0.004 |
|  |  | Act(Low):Pred(Lion) | -0.01 | 0.02 | -0.17 | 0.865 |
|  |  | UI:Act(Low):Pred(Lion) | -0.14 | 0.03 | -4.14 | <0.001 |
